# The Articulating Frame

**Published:** 1906-09

**Authors:** Stewart J. Spence

**Affiliations:** Chattanooga, Tenn.


### THE ARTICULATING FRAME.

Stewart J. Spence, Chattanooga, Tenn.

It is an error to suppose that any thing in the way of an articulator is good enough, or that the model may be placed on the frame in any sort of way. The fact is, that no articulating frame thus far made is good enough, for none of them has any device for adjusting its width to that between the patients' condyles, nor any method for holding to place its jaws when swung sideways or drawn forward. As for the old-fashioned articulator that has only the up and down motions, it is simply not worth talking about.

A model may be placed wrongly on an articulator in various ways,—thus: (1) by being placed too far forward or too far back; (2) by being placed so that the plane of occlusion of the teeth will not be directed to the joint; (3) by being placed askew, so that its heels are not equidistant from the joint adjoining each; (4) and by being tilted, so that the left side is higher proportionately than the right side or *vice versa.* And lastly, the error may be in a combination of one or more of these four mal-positions.

The face-bow of Dr. Snow is a good device for obtaining correct position. If used, however, with an articulator which has its joint midway between its upper and lower jaws (as the Kerr) the measurement should be taken not from the condyle itself, but from that point which may be termed the true joint of the jaw; which is located about 1¼ inches perpendicular to the condyle and about a half inch posterior to the ramus. Thus this imaginary joint is located in space. This fact makes it a little difficult to take its measurement.

The writer rarely uses the face-bow, because it somewhat interferes with other things in taking the bite, but instead takes the measurement from joint to position of lower incisors by a divider, when desired. As, however, it is not important to get this distance if the bite is not to be opened or closed any on the articulating frame, and as the distance between the condyles of frame cannot, as before remarked, be adjusted to different widths, and as to leave them, say, 4 inches wide while reducing the forward position of lower incisor point to, say, three and a half inches from joint, interferes seriously with results in the sideward bite, I deem it best usually to simply position the incisor point at four inches. If a little care is taken in placing the model on the frame the other three mal-positions are easily avoided. It is well to use a plaster that sets slowly enough to give the operator time to see that he is getting things right, and to shift his model and accompanying wax bite until they are so.

The operator must not think that it matters not whether his articulator's jaw are parallel or, on the contrary, at an angle to each other, for frames are manufactured so that when the jaws are parallel the path of the condyle is at the angle usually correct, and therefore if the upper jaw of the frame is thrown up to admit an unusually bulky model the said condyle path is altered accordingly. This fact, however, may be taken advantage of when it is desired to give the condyle's path an unusual inclination. One articulating frame (the Kerr) has a special device for altering said inclination of the path.

If the "true joint" of the human jaws be one-and-a-quarter inches perpendicular to the condyle it follows that said condyle will, in moving forward and downward along its path, pursue an arc of a circle of two-and-a-half inches diameter, and having said true joint as center. It is easy to see by testing with dividers whether or no the path of an articulating frame has this inclination.

All articulating frames having their joint on the same level as their upper jaws will give false results in the sideward bite, the incisors occluding earlier than the posterior molars by about 1-32 inch.

This leaves the Kerr the only articulator on the market correct in this particular. I regret to say, however, that this otherwise excellent frame has a measurement of only a little over three and a half inches from condyle to condyle, instead of the average width of the human jaw, which is four inches. However, this error in the frame may be corrected by shifting some of its machinery,—placing the condyle paths outside, instead of inside, the uprights to which they are attached by the thumbscrew used for adjusting the inclination of the condyle paths.

As it is often very desirable that the jaw of a frame be maintained in either the protruded or sideward position for a sufficient length of time to give the operator full opportunity to observe how his molars are articulated, as seen from behind, and as it is difficult to hold the frame in this position by the fingers, I recommend the dentist to add to his frame two little screws, so placed that by turning in both of them the lower jaw of the frame may be thrust forward to the position of the protruded bite, and by turning in only one screw the jaw may be held in the position of the sideward, or masticatory, bite. These little screws also answer another purpose, viz.. that if on removing the wax bite it becomes evident that the patient bit a little too much sideways in making the bite, by means of one of these screws the jaw can be moved sideward to correct position. Somebody ought put on the market a perfect articulatory frame, adjustable in all particulars.

In all except full upper and lower cases, that is, whenever teeth remain to be duplicated by plaster in the articulators, it is well to use Spence's plaster for this purpose, because a tooth of plaster of Paris, being soft, can be worn and compressed during the time of setting up a denture sufficiently to injure the subsequent occlusion.

